# Results from a natural experiment: initial neighbourhood investments do not change objectively-assessed physical activity, psychological distress or perceptions of the neighbourhood

**DOI:** 10.1186/s12966-019-0793-6

**Published:** 2019-03-27

**Authors:** Tamara Dubowitz, Madhumita Ghosh Dastidar, Andrea S. Richardson, Natalie Colabianchi, Robin Beckman, Gerald P. Hunter, Jennifer C. Sloan, Alvin K. Nugroho, Rebecca L. Collins

**Affiliations:** 1 RAND Corporation, 4570 Fifth Avenue, Suite 600, Pittsburgh, PA USA; 2 RAND Corporation, 1776 Main Street, Santa Monica, CA USA; 3 University of Michigan, School of Kinesiology, Ann Arbor, MI USA

**Keywords:** Physical activity, Environment, Intervention, Low-income neighborhood, Natural experiment, Difference-in-difference, Psychological distress, Neighbourhood perceptions

## Abstract

**Background:**

Few studies have assessed objectively measured physical activity (PA), active transportation, psychological distress and neighborhood perceptions among residents of a neighborhood before and after substantial improvements in its physical environment. Also, most research-to-date has employed study designs subject to neighborhood selection, which may introduce bias in reported findings.

We built upon a previously enrolled cohort of households from two low-income predominantly African American Pittsburgh neighborhoods, matched on socio-demographic composition including race/ethnicity, income and education. One of the two neighborhoods received substantial neighborhood investments over the course of this study including, but not limited to public housing development and greenspace/landscaping. We implemented a natural experiment using matched intervention and control neighborhoods and conducted pre-post assessments among the cohort. Our comprehensive assessments included accelerometry-based PA, active transportation, psychological distress and perceptions of the neighborhood, with assessments conducted both prior to and following the neighborhood changes. In 2013, we collected data from 1003 neighborhood participants and in 2016, we re-interviewed 676 of those participants. We conducted an intent to treat analysis, with a difference-in-difference estimator using attrition weighting to account for nonresponse between 2013 and 2016. In addition, we derived an individual-level indicator of exposure to neighbourhood investment and estimated effect of exposure to investment on the same set of outcomes using covariate-adjusted models.

**Results:**

We observed no statistically significant differences in activity, psychological distress, satisfaction with one’s neighborhood as a place to live or any of the other measures we observed prior to and after the neighborhood investments between the intervention and control neighborhoods or those exposed vs not exposed to investments.

**Conclusions:**

Using this rigorous study design, we observed no significant changes in the intervention neighborhood above and beyond secular trends present in the control neighborhood. Although neighborhood investment may have other benefits, we failed to see improvement in PA, psychological distress or related outcomes in the low-income African American neighborhoods in our study. This may be an indication that improvements in the physical environment may not directly translate into improvements in residents’ physical activity or health outcomes without additional individual-level interventions. It is also possible that these investments were not dramatic enough to spur change within the three year period. Additional studies employing similar design with other cohorts in other settings are needed to confirm these results.

**Trial registration:**

Trial Registration is not applicable since we did not prospectively assign individuals to a health-related intervention.

## Background

In the United States (U.S.), racial and socioeconomic segregation has created unequal access to opportunity with both acute and cumulative impacts. Neighbourhood aesthetics, safety, and access to and quality of services (childcare, education, retail, etc.) may ultimately translate into resident health. This has been brought to light in at least a decade’s worth of public health literature [1–5].

Regular physical activity (PA) is also known to contribute to positive health outcomes, including lower incidence of cardiovascular diseases, diabetes, depression, certain cancers, and obesity. Increasing evidence suggests that there are associations between neighbourhood features that are conducive to PA (e.g., parks, trails, PA facilities, safety) and engagement in PA [6–10]. Such neighbourhood resources, sometimes referred to as the Physical Activity Environment, have been shown to be less plentiful in neighbourhoods with low socioeconomic status residents and/or a high percentage of racial/ethnic minorities [11, 12]. In addition, neighbourhood physical and social characteristics from housing, landscaping, and sidewalk conditions to social cohesion and employment have also been shown to predict health and well-being as well as PA and active transportation [13–18]. Thus, investing in the neighbourhood environment, particularly in low income or racially/ethnically isolated neighbourhoods, has been deemed a promising strategy to potentially improve resident health, including physical activity, mental health and related outcomes.

Yet much of the available evidence on associations between neighbourhood characteristics, the PA environment and resident activity has been cross-sectional, [9, 19–21] and few studies [22–27] have assessed health outcomes, including PA, on the same residents both before and after a substantial change in the physical environment. Thus, it is still unclear whether self-selection biases are responsible for the significant associations between features of the environment and health outcomes, or whether there are specific neighbourhood characteristics that may lead (causally) to health or health behavior improvements. Further, much of the current evidence base around physical activity has employed self-reported measures [28]. Given the awareness of biases in self-reported data, [29–31] it is important to establish a body of evidence that relies on objective assessments.

**Fig. 1:**
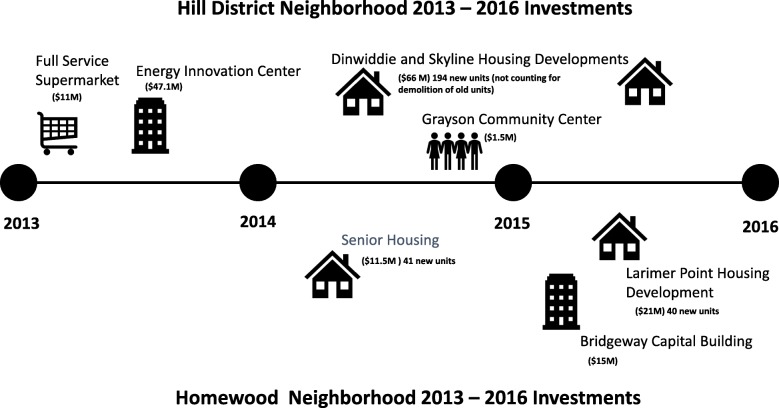
Neighborhood Investments 2013–2016 Total Development Cost. Intervention and comparison neighborhoods and investments between 2013 and 2016 in each. During the study period (October 2013 through May 2016), the Hill District received $193,628,994 in investments including a full-service grocery store, public housing development, park and greenspace renovations, and an energy innovation center dedicated to workforce development and incubation of businesses. During this same period, Homewood investments totaled $47,516,268 and were mostly in housing

Natural experiments fill a critical gap in research design [24, 32, 33]. In place of randomizing place-based changes (which could be either unethical, impractical or impossible) or randomly assigning residents to move (which can introduce stress and other confounds), observing individuals before and after naturally occurring place-based changes can give an approximation of the impact of these changes on individuals. Simultaneously observing a geographically and socio-demographically equivalent sample at baseline allows for the control of secular shifts (those changes that occur without intervention). Planned commercial, housing and greenspace investments that would change the streetscape and physical environment in a lower-income, African American neighbourhood in Pittsburgh, PA, and not in a nearby community that was otherwise similar, provided the opportunity for such an experiment. During the study period (October 2013 through May 2016), the Hill District neighborhood (intervention) received a total of $193,628,994 in investments including a full-service grocery store, multiple public housing developments, a community center, and an energy innovation center dedicated to workforce development and incubation of businesses. These investments also changed the streetscape surrounding the developments, providing improved aesthetics (e.g. trees, grass) and walkability (e.g. sidewalks, street crossings). During this same period, Homewood (comparison neighborhood) also received investments, almost exclusively in housing developments. These totaled $47,516,268, far less than what was observed in the intervention neighborhood. We illustrate the investments that happened in each of the neighorhoods, in Fig. 1.

Consistent with the prior cross sectional studies that have linked neighborhood characteristics such as landscaping and sidewalks to physical activity, [6–10] we hypothesized that following the completion of these improvements, residents of the Hill District neighborhood would experience increases in physical activity, active transportation and psychological precursors to physical activity, increases in positive neighbourhood perceptions and satisfaction, and decreases in psychological distress relative to residents of Homewood observed over the same time period. Specifically, we expected that the improved aesthetics and walkability of the residential environment (i.e. the new buildings, sidewalks, plantings, and street crossings) would reduce perceived barriers to PA and shift intentions, spur residents to walk to the new grocery and to existing retail venues (increasing active transportation), and motivate them to walk more often for exercise or leisure to explore and enjoy the new areas. We also hypothesized that these improvements would improve residents’ well-being and their perceptions of and satisfaction with their neighborhood. In contrast, we expected little if any change among residents of the comparison neighborhood, Homewood, where physical changes were less substantial.

## Methods

The Pittsburgh Research on Neighbourhoods, Exercise and Health (also called ‘PHRESH Plus’) was built upon an earlier study (PHRESH), which began in 2011, and was designed to examine the impact of the opening of a full-service supermarket in the Hill District on resident diet and food shopping behaviors [34–36]. In 2011, the Hill District and Homewood neighbourhoods were socio-demographically and geographically matched. The Hill District was 1.37 mile^2^ (population of 10,219), while Homewood was 1.45 mile^2^ (population of about 8300). Residents of both neighbourhoods were predominantly African-American with median household income less than $15,000 [37, 38].

**Fig. 2:**
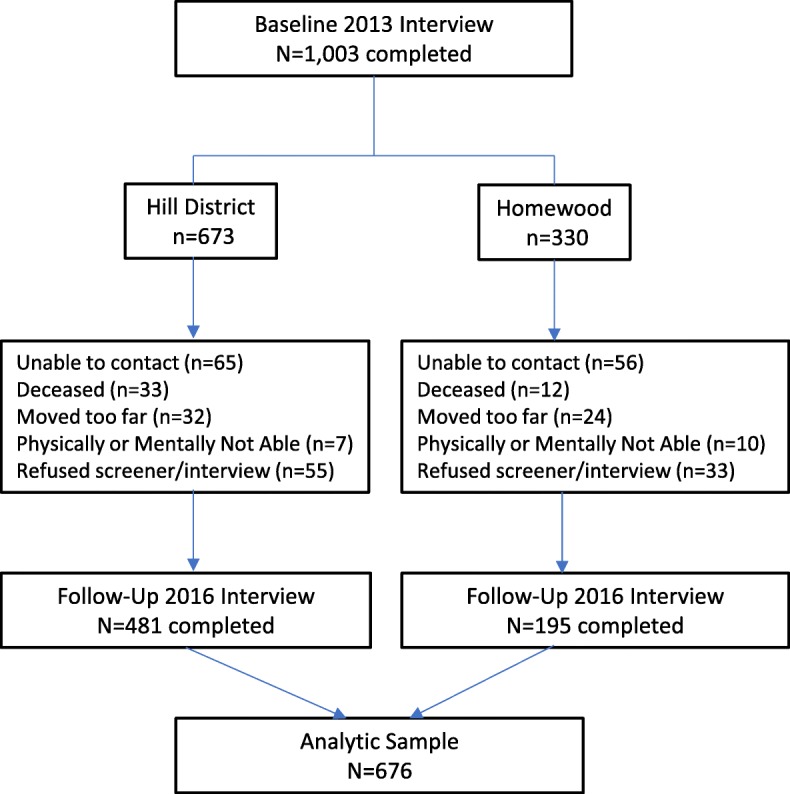
Derivation of Analytic Sample. This shows the derivation of the intervention and comparison neighborhoods’ baseline (2013) and follow-up (2016), and the analytic sample used

The sampling strategy for the enrolled cohort from the two neighbourhoods is described elsewhere [34–36]. Briefly, the original PHRESH study drew a stratified random sample of residential addresses in the two study neighborhoods, from a master list of addresses obtained by merging Allegheny County Office of Property Investment data with the Pittsburgh Neighbourhood and Community Information System. By design, households in the intervention neighbourhood (Hill District) were oversampled; 897 were enrolled in the intervention and 475 in the control neighborhood (Homewood) for a total sample of 1372 in 2011. In 2013, prior to the supermarket opening and the PHRESH follow-up, PHRESH Plus re-surveyed 1051 (response rate of 77%) of the original study participants, collecting PA and related measures for the PHRESH Plus baseline. Of the 1051, 1003 still lived within the boundaries of the study neighborhoods in 2013 and were included in this study. At the PHRESH Plus follow-up assessment in 2016, PHRESH Plus interviewed 676 of those 1003 households (76% response rate). Our analytic sample includes these 676 cases [i.e., those with both a baseline (2013) and follow-up (2016) interview and still residing in one of the two neighborhoods]. We have provided a breakdown of the sample by group in Fig. 2.

Participants responded to a 60 min survey at each wave, using interviewer-administered computer assisted personal interviewing, had their height and weight measured, and then wore an Actigraph GT3X+ accelerometer for 7 days. Survey participants received an incentive of $25 for completing a survey and up to $50 in addition for completing all 7 days of accelerometry. Study protocols were approved by RAND Institutional Review Board (IRB).

### Measures

Our primary outcome of interest was objectively measured average daily minutes of physical activity. All participants were given a tri-axial Actigraph GT3X+ accelerometer to wear on their non-dominant wrist for 7 consecutive (24 h) days. Data were processed in R using the GGIR package 1.2–8 (http://cran.r-project.org) and using static periods in the data, calibration error was estimated and corrected if necessary. The wrist-mounted GT3X+ has been found to be a reliable and valid method for measuring physical activity [39, 40]. The 100 mg threshold used in the study to define MVPA was derived from a validation study that included a wide age range of adults and compared accelerometry data against indirect calorimetry [39]. A calibration algorithm available in GGIR was utilized to correct for any bias due to an inaccurately calibrated sensor. This is achieved by examining the vector magnitude during periods of non-movement (which should register at 1 *g* due to the gravitation component in the signal) and making corrections in the data if needed [41]. Our team identified nonwear time, [42, 43] and quantified acceleration. Nonwear time was classified when either of the following conditions were present: 1) the standard deviation (SD) was less than 13 mg for 2 of the 3 axes or 2) if the value range of each accelerometer axis was less than 150 mg, calculated for moving windows of 60 min with 15 min increments. The non-wear criteria used in the study were based on a validation study, [43] and have been used in other studies [42, 44]. Minutes of moderate to vigorous physical activity (MVPA) were defined as a bout of at least 10 min of activity above the 100 mg threshold, [39] where at least 80% of the bout was above the threshold of 100 mg. The *average daily minutes of MVPA* was calculated for those with valid wear time, set to be at least 10 h of wear on 4 or more days.

To capture *active transportation*, we used an individual question from the International Physical Acitivty Questionnaire (IPAQ), which has found acceptable reliability and validity in previous samples [45, 46]. It asked, “Now think (only) about the walking you might have done to travel to and from work, to do errands, or to go from place to place; do not include walking that you have done solely for recreation, sport, exercise, or leisure.” Respondents indicated number of days walked during the last 7 days, and time usually spent walking on those days. Minutes walking per week was computed.

We captured *psychological distress* with the Kessler 6 (K6) scale. Participants reported the frequency with which they experienced six distress symptoms (e.g., “feeling hopeless”) in the last 30 days. Responses were provided on a five-point scale and summed, severe distress = 13+ points [47]. The K6 instrument is well validated, and is strongly associated with demographic and socioeconomic status [48, 49].

*Neighbourhood satisfaction* [50] was assessed by asking, “All things considered, would you say you are very satisfied, satisfied, dissatisfied, very dissatisfied or neutral – neither satisfied nor dissatisfied, with your neighbourhood as a place to live?” Others have used this single item question to understand residents’overall perceptions of their neighborhood [51–54]. This item was dichotomized with the highest two levels (satisfied or very satisfied coded as 1, and neutral, dissatisfied or very dissatisfied coded as 0) .

*Other measures.* Height was measured to the nearest eighth inch using a carpenter’s square and an 8-ft folding wooden ruler marked in inches. The weight of each participant was measured to the nearest tenth of a pound using the SECA Robusta 813 digital scale. Body mass index (BMI) was calculated as the ratio of objectively-measured weight (kg) divided by squared height (m^2^). We created two dichotomous outcomes, *overweight* (defined as BMI > = 25) and *obese* (defined as BMI > = 30).

To measure *perceived infrastructure,* we used five items from the Neighborhood Enviornment Walkability Scale – Abbreviated version (NEWS-A), specifically the Infrastructure subscale. Participants rated aspects of their neighbourhood including sidewalks, lighting and crosswalks with five questions containing 5-point scale response choices ranging *from strongly disagree to strongly agree* [55]. *Perceived aesthetics,* also from NEWS-A, assessed the perceived presence of trees, interesting neighbourhood features and the attractiveness of the neighbourhood environment. *Perceived safety* tapped into how safe participants felt in their neighbourhood during the day and evening, and how much of a problem they perceived crime and violence to be. This perceived safety measure has demonstrated acceptable to good internal consistency [56, 57]. *Social cohesion* assessed the level of perceived neighbourhood interconnectedness, trust, and shared values. Social cohesion has been tested to have strong internal consistency [56, 57]. We measured *access to services* [55] and *traffic along nearby streets* [58].

We also assessed psychological precursors to physical activity, all of which have been previously developed and validated [59, 60]. These included *social norms* (e.g., “How often do your friends or family participate in physical activity such as walking, jogging, bicycling, or playing sports?”); [61] *intentions to engage in PA*, (“Do you intend to engage in physical activity three or more times a week for at least 10 minutes at a time during the next year?”) [62–64]. *Barriers to PA* (eight items [65] with a 5-point response scale). *Self-efficacy to engage in PA* over the next 6 months in the face of a variety of barriers (10 items with a 10-point response scale) [66]. To create a measure of *PA outcome expectancies*, participants were asked whether they agreed (on five 5-point response scales) that if they participate in physical activity they will experience a variety of positive outcomes (e.g., “feel less depressed”, lose weight) [67–69]. Responses were averaged.

We included individual-level *sociodemographic variables* to use as covariates, including age, gender, marital status, income, a binary indicator of whether there were any children living in the household, and level of education.

Finally, physical functioning, which is a 10-item sub-scale part of the SF-36 (36-Item Short Form Survey Instrument which taps into self-reported health) [70, 71] measures the extent to which health limits physical activities. The ten items ask about the extent to which health limits you from doing daily activity including bathing, dressing, walking, bending or kneeling, climbing stairs, lifting or carrying groceries, and doing moderate or vigorous activities. In the analyses presented here, we test for changes in PA using only the subsample of individuals whose physical functioning scores indicated that they had at least moderate levels of physical functioning, defined as a scale score at one standard deviation below the mean or greater. Other analyses use the full sample of 676 individuals.

### Statistical analyses

We examined comparability of baseline (2013) characteristics of the randomly sampled households in the two neighbourhoods and tested for statistically significant differences with t-tests and chi-squared tests. Next, for each of the outcomes described above, we computed (i) the average difference between baseline and follow-up values in the intervention group, (ii) the average difference between baseline and follow-up values in the comparison group, and (iii) a difference-in-difference estimator indicating changes in the intervention group over time compared with those in the comparison group. Each value was tested to determine if it was significantly different from zero. The primary analysis employed an *intention-to-treat* approach, [72, 73] comparing differences in average outcomes for the entire intervention neighbourhood against those observed in the comparison neighbourhood (regardless of whether they moved during the follow-up period). All analyses were adjusted for sociodemographics.

One challenge with natural experiments is that researchers cannot control whether exposure to the “intervention” (in this case, neighbourhood improvements in housing, greenspace and commercial development) is implemented as planned in the intervention neighborhood and completely absent in the comparison. At the time our study was conceived and designed investments and development were planned for only one neighborhood (the Hill District). However, as noted in our introduction, some investments and development occurred in the comparison neighborhood (Homewood) as well. This raised the possibility that more substantial changes than the secular shifts we expected might occur in Homewood. Although we still expected greater change in the Hill District than Homewood, we reasoned that our difference in difference design might not be sensitive enough to detect a statistical difference in the relative size of these changes. To guard against Type II error, we supplemented our neighborhood level difference in difference analysis with an individual-level approach. In it, we designated study participants, *regardless of neighborhood*, as exposed or unexposed to investments based on their proximity to a new investment or development that occurred in either their own neighborhood or the other neighborhood under study. That is, instead of comparing ‘intervention’ and ‘control’ neighborhoods, we compared study participants living within a specified distance from a community investment/development to those who lived further away, no matter which neighborhood the participants lived in.

To create this individual-level indicator of exposure to neighbourhood investments we coded a household as ‘close to an investment’ (=1) if the household was within one-tenth of a mile of any neighbourhood investment project that occurred after baseline data collection and before follow-up data collection, and coded as ‘further from investment’ (=0) otherwise. We then ran an additional difference-in-difference analysis comparing these two groups of individuals. To correct for pre-existing differences between the two groups, we controlled for sociodemographics in this analysis, as well.

Analyses were performed using Proc Genmod in the statistical software SAS, version 9.3, accounting for correlations among repeated measurements of each participant. In our models, we assume an autoregressive correlation structure. Analyses were weighted to account for sample attrition between baseline and follow-up to ensure that results generalize to the baseline sample, derived as the inverse probability of response at follow-up, estimated using a logistic regression model with socio-demographics and additional baseline characteristics as predictors. Due to the large number of significance tests conducted in our analyses, there was risk of inflated type I error. We therefore adjusted for multiple testing using the Benjamini-Hochberg (B-H) approach [74].

## Results

Table 1 shows characteristics of the analytic cohort (i.e., participants present at both baseline and follow-up and still living in the study neighborhoods) in the intervention and control neighbourhoods. We saw very similar sociodemographic characteristics. In both neighbourhoods, 96% of the sample were African American, and just over 81% had a per capita household income below $20,000/year. Only a quarter or less were married or living with a partner, while about half had the education equivalent to a high school diploma or less.

**Table 1:** Baseline Characteristics of Analytic Sample (*n* = 676) - Mean (SD) or Percent

Characteristic	Hill District (intervention neighborhood); Percent, Mean (SD) (*n* = 481)	Homewood (comparison Neighborhood); Percent, Mean (SD) (*n* = 195)
Race/Ethnicity		
African American or Black	95.7	95.8
Other	4.3	4.2
Age		
18–34	13.9	13.0
35–44	10.3	14.3
45–54	23.0	25.5
55–64	24.3	20.9
65–74	16.0	18.3
75+	12.5	8.1
Mean Age	54.8 (16.4)	53.3 (15.5)
Gender		
Male	20.7	26.6
Female	79.3	73.4
Per Capita Annual HH Income		
< $5000	23.0	30.5
$5000 - $9999	25.3	24.4
$10,000 - $19,999	33.2	26.4
$20,000+	18.5	18.8
Marital Status		
Married/living with partner	19.0	25.7
Never married	42.8	35.1
Widowed/divorced/separated	38.1	39.2
Educational attainment		
< High school diploma	12.9	10.2
High school diploma	41.7	35.7
Some college/technical school	32.5	35.4
College degree	12.9	18.8
Any Children in Household	25.7	32.0
Own or borrow a car	58.6	57.6
Physical Functioning	66.65 (28.8)	66.06 (29.7)

**source** Authors’ calculations. *HD* = Hill District, *HW* Homewood, *PA* Physical Activity; All results include weighting to adjust for sample attrition between baseline (2013) and follow up (2016)

Table 2 shows results of our primary analysis, the neighborhood level (intent to treat) difference- in-difference comparison of PA, distress, and other health outcomes, perceptions of and satisfaction with the neighborhood, and psychological precursors to PA. For tests significant after the B-H adjustment, *p* values are bolded. As shown in the table, we observed no significant difference in differences among any of our outcomes. That is, there were no changes over time in one neighbourhood that proved significantly different from changes over time in the other neighbourhood. However, there were some significant changes *within* each of the neighbourhoods.

**Table 2:** Changes in Physical Activity, Neighborhood Environment and Social Norms for Study Participants From Baseline to Follow-Up, By Neighborhood

	Intervention (Hill District)			Comparison (Homewood)			Difference-in-Difference	
	Baseline Percent, Mean (SE) (*n* = 481)	Change ^+^ Percent, Mean (SE) (*n* = 481)	*p*-value	Baseline Percent, Mean(SE) (*n* = 195)	Change ^+^ Percent, Mean (SE) (*n* = 195)	*p*-value	HD Change - HW Change (*n* = 676)	*p*-value
Health Outcomes								
Daily MVPA in minutes for those participants who were physically functional	6.89 (0.90)	− 0.83 (0.80)	0.299	6.18 (1.22)	− 1.06 (1.36)	0.435	0.24	0.813
Self-reported average min/week walking place to place for those participants who were physically functional	197.74 (15.02)	20.22 (20.63)	0.327	201.93 (25.71)	−16.18 (25.44)	0.525	36.40	0.270
Body Mass Index (kg/m^2^)	30.73 (0.32)	−0.43 (0.16)	0.009	31.68 (0.67)	−0.64 (0.27)	0.015	0.22	0.487
Obese (% with BMI > =30)	49.17	−2.89	0.072	53.57	−2.95	0.289	0.06	0.983
Overweight or Obese (% with BMI > =25)	79.46	−2.35	0.090	79.30	−3.78	0.047	1.43	0.568
Psychological Distress (K6)	4.23 (0.20)	−0.00 (0.20)	0.99	4.84 (0.35)	−0.22 (0.33)	0.505	0.22	0.587
Neighborhood Environment								
Perceived Infrastructure (e.g. sidewalks, lighting, crosswalks, pedestrian signals) (5 point scale)	3.28 (0.03)	0.22 (0.03)	**<.0001**	3.06 (0.05)	0.14 (0.06)	0.024	0.08	0.238
Aesthetics (e.g. trees, interesting things, attractive sights) (5 point scale)	3.00 (0.04)	0.22 (0.04)	**<.0001**	2.48 (0.07)	0.26 (0.07)	**<.0001**	−0.04	0.575
Safety (5 point scale)	3.03 (0.03)	0.15 (0.03)	**<.0001**	2.55 (0.06)	0.23 (0.06)	**0.0002**	−0.08	0.280
Satisfaction with one’s neighborhood as a place to live (% satisfied or very satisfied)	69.49	3.89	0.117	42.64	9.78	0.024	−5.89	0.342
Social Cohesion	3.11 (0.04)	0.13 (0.04)	**0.0005**	2.96 (0.07)	0.21 (0.06)	**0.001**	−0.08	0.296
Many places in easy walking distance of home (% who agree or strongly agree)	45.28	21.86	**<.0001**	33.06	12.89	**0.004**	8.97	0.107
Traffic along nearby streets makes it difficult or unpleasant to walk (% who agree or strongly agree)	27.94	−6.24	**0.014**	34.68	−4.81	0.301	−1.43	0.648
Social Norms and Environment								
How often do your friend and family participate in physical activity (PA)?	2.92 (0.05)	−0.06 (0.06)	0.363	2.93 (0.08)	0.26 (0.12)	0.040	−0.31	0.024
How often do you see people in your neighborhood participating in PA?	3.20 (0.06)	0.09 (0.07)	0.221	3.21 (0.10)	−0.03 (0.10)	0.743	0.12	0.335
High intentions to engage in PA (% who intend)	60.19	−5.35	0.053	60.98	−7.26	0.116	1.91	0.723
High barriers to PA (%)	12.09	−5.98	**0.0004**	28.63	−19.57	**<.0001**	13.59	0.093
Self-efficacy to engage in PA	5.34 (0.11)	−0.03 (0.12)	0.808	5.67 (0.17)	0.16 (0.17)	0.341	−0.19	0.358
Outcome expectancies	3.98 (0.03)	−0.03 (0.04)	0.351	3.96 (0.05)	−0.05 (0.05)	0.336	0.02	0.792

**source**: Authors’ calculations. **Notes** Results bolded in the table are significant after Benjamini-Hochberg multiple testing adjustment at the 5% significance level; ^+^ Change is computed as difference between follow up and baseline; *PA* Physical Activity. All models are covariate adjusted for sex, age, education, income, marital status, and any children, and include attrition weights

First, we saw pre B-H adjustment decreases in BMI across both neighbourhoods. Specifically, the decrease in BMI units was .43 in the intervention neighborhood and .64 in the comparison neighborhood.

In addition, within each of the neighbourhoods, we observed significant differences between baseline and follow-up in perceptions of aesthetics (e.g., trees, attractive sights), safety, social cohesion and places that are in easy walking distance from home. In the intervention neighbourhood, we also saw improvements in perceived infrastructure (e.g., sidewalks, lighting, crosswalks) and perceptions around traffic that makes it unpleasant to walk. We also observed statistically significant decreases in both neighbourhoods of reported barriers to PA.

Table 3 shows the individual level (i.e., regardless of neighborhood) difference-in-difference comparison of participants living ‘close to investment’ versus participants ‘further from investment,’ with similarly null results regarding ‘exposure to investment.’ With this distance to investment difference-in-difference analysis, we observed two pre- B-H adjustment difference in differences: BMI and reported places within easy walking distance to home. For both of these outcomes, there was increased improvement (i.e., decrease in BMI and improvement in reported places to walk to) for those participants who lived ‘closer to’ an investment.

**Table 3:** Changes in Physical Activity, Neighborhood Environment and Social Norms for Study Participants From Baseline to Follow-Up, By Distance from Neighborhood Investment

	Intervention (Within .1 mile of Investment)			Comparison (Further than .1 mile from Investment)			Difference-in-Difference	
Variables	Baseline Percent, Mean (SE) (*n* = 119)	Change ^+^ Percent, Mean (SE) (*n* = 119)	*p*-value	Baseline Percent, Mean(SE) (*n* = 554)	Change ^+^ Percent, Mean (SE) (*n* = 554)	*p*-value	Intervention Change – Comparison Change (*n* = 673)	*p*-value
Health Outcomes								
Daily MVPA minutes for those participants who were physically functional	7.34 (2.32)	−2.18 (1.50)	0.148	6.55 (0.76)	−0.71 (0.80)	0.371	−1.47	0.318
Self-reported average min/week walking place to place for those participants who were physically functional	139.0 (20.4)	−2.42 (26.53)	0.927	209.6 (14.7)	11.31 (18.71)	0.545	−13.74	0.738
Body Mass Index (kg/m^2^)	32.13 (0.70)	−1.16 (0.35)	**0.0008**	30.75 (0.31)	−0.35 (0.15)	0.020	−0.81	0.032
Obese (% with BMI > =30)	58.56	−1.99	0.563	48.60	−3.11	0.043	1.12	0.777
Overweight (% BMI > =25)	78.89	−4.38	0.021	79.65	−2.45	0.059	−1.93	0.443
Psychological Distress (K6)	4.83 (0.46)	−0.38 (0.38)	0.316	4.30 (0.19)	0.04(0.19)	0.850	−0.42	0.331
Neighborhood Environment								
Perceived Infrastructure (e.g. sidewalks, lighting, crosswalks, pedestrian signals) (5 point scale)	3.30 (0.06)	0.23 (0.06)	**.0003**	3.20 (0.03)	0.19 (0.03)	**<.0001**	0.04	0.564
Aesthetics (e.g. trees, interesting things, attractive sights) (5 point scale)	2.97 (0.08)	0.35 (0.09)	**<.0001**	2.82 (0.04)	0.21 (0.04)	**<.0001**	0.15	0.123
Safety (5 point scale)	2.83 (0.06)	0.13 (0.07)	0.043	2.90 (0.03)	0.18 (0.03)	**<.0001**	−0.05	0.523
Satisfaction with one’s neighborhood as a place to live (% satisfied or very satisfied)	68.41	8.75	0.125	60.82	5.31	0.033	3.44	0.486
Social Cohesion	3.11 (0.06)	0.07 (0.06)	0.251	3.05 (0.04)	0.17 (0.04)	**<.0001**	−0.09	0.198
Many places in easy walking distance of home (% who agree or strongly agree)	46.23	33.76	**<.0001**	40.08	17.19	**<.0001**	16.57	0.005
Traffic along nearby streets makes it difficult or unpleasant to walk (% who agree or strongly agree)	32.82	−4.75	0.363	29.30	−6.15	**0.014**	1.40	0.736
Social Norms and Environment								
How often do your friend and family participate in physical activity (PA)?	2.98 (0.11)	0.12 (0.14)	0.390	2.91 (0.05)	0.01 (0.07)	0.931	0.11	0.461
How often do you see people in your neighborhood participating in PA?	3.18 (0.12)	0.23 (0.16)	0.150	3.21 (0.06)	0.01 (0.06)	0.869	0.22	0.20
High intentions to engage in PA (% who intend)	56.33	−5.26	0.300	61.44	−6.43	0.016	1.17	0.819
High barriers to PA (%)	18.76	−12.05	0.008	15.61	−9.18	**<.0001**	−2.87	0.716
Self-efficacy to engage in PA	5.07 (0.22)	0.11 (0.24)	0.650	5.51 (0.10)	−0.00 (0.11)	0.996	0.11	0.677
Outcome expectancies	3.95 (0.06)	−0.01 (0.07)	0.877	3.98 (0.03)	−0.04 (0.03)	0.189	0.03	0.644

**source**: Authors’ calculations. **Notes** Results bolded in the table are significant after Benjamini-Hochberg multiple testing adjustment at the 5% significance level; ^+^ Change is computed as difference between follow up and baseline; *HD* Hill District, *HW* Homewood, *PA* Physical Activity, *NBHD* Neighborhood. All models are covariate adjusted for sex, age, education, income, marital status, and any children, and include attrition weights

We observed improvement in both groups in infrastructure, aesthetics, and reported places within easy walking distance. In the ‘further from investment’ group, we saw a statistically significant improvement in safety, improvement in traffice and social cohesion. Finally, we observed a statically significant decrease in barriers to engaging in physical activity among the ‘further from investment’ group.

The ‘closer to investment’ group demonstrated a significant decrease in BMI and although the “further from” investment group also experienced a decrease, it was not (post B-H) statistically significant.

## Discussion

This quasi experimental study is one of the few United States-based studies that has been able to compare residents and their health behaviors over time in an intervention and control neighbourhood, in order to examine potential impacts of neighbourhood investments on physical activity, psychological distress, perceptions of the neighbourhood, and related health outcomes. It is also one of the only studies, to our knowledge, that has objectively and longitudinally measured physical activity through accelerometry in a predominantly African American low-income cohort.

In both of our difference-in-difference analyses (i.e., the neighbourhood intent to treat analysis and the individual level ‘closer to or further from investment’ analysis), we found no statistically significant differences in PA, psychological distress, perceptions of the neighbourhood environment, or psychological precursors to physical activity, between the changes that occurred in the intervention neighbourhood compared with the comparison neighbourhood – or between participants closer to investments compared with those participants who lived further from investments.

With our distance-to-investment difference-in-difference analysis, we observed two pre- B-H adjustment difference in differences: BMI and reported places within easy walking distance to home. We observed a .8 unit decrease in BMI for those participants who lived within 0.1 mile of an investment compared to decreases among participants who lived further away. In addition, for those participants who lived within 0.1 mile of an investment compared to participants who lived further away, we observed a 17% greater increase in reports of having many places within easy walking distance of home. Although these changes are consistent with hypotheses, their failure to reach significance after adjustment for multiple testing and the lack of consistency with the intent to treat results are not.

Generally, our results indicate that the neighbourhood changes that we considered did not affect physical activity or its psychological precursors, perceptions of neighbourhoods, or mental distress. Effects on BMI are less clear, but we could not confirm them statistically. Several factors should be kept in mind, however. It is possible that the investments studied were not substantial enough to elicit observable changes. In addition, we examined these changes over a relatively short time period (3 years); this may be insufficient given the nature of how neighbourhood development and investment may ultimately impact health and health behaviors.

In addition, our cohort is older and sedentary. We note the very low MVPA at baseline. About 7 min of MVPA (average) daily is far beneath the 150 min/week recommendation. Given this, it is possible that neighbourhood improvements alone would not be sufficient to increase the MVPA of this cohort. Given these factors, it will be important for others to replicate these findings in other settings, with other cohorts, with the ability to look at other investments, using similarly rigorous designs.

Although we used accelerometry to objectively capture activity, we did not use any additional measures (e.g., GPS) to determine *where* the activity was occurring. Yet we believe that asking participants to allow GPS location tracking would not have been feasible in this population. Issues from devices being too similar to “house arrest” trackers to mistrust of researchers, were just some of the obstacles we faced. If changes in activity within the neighborhoods were small compared to participants’ activity outside of neighborhoods, our general measure of physical activity regardless of location might have missed this change. We did, however, ask (at both baseline and follow up) where participants went most often to engage in physical activity, how often participants walked *in their neighborhood* either to get somewhere or for physical activity, and how often they visit parks in their neighborhood. Looking at these neighborhood-specific questions, there were also no statistically significant difference in differences.

There have been numerous calls for the assessment of the impact of changes in policies, neighborhoods, and other structural changes to combat obesity and obesity-related health behaviors. Interpreting results from such assessments is often complicated, in part because focusing on one particular policy (or feature of the built environment) may not yield results because of the complex factors that ultimately play into both health and health behaviors, and the difficulty of measurement of potential change. Other natural experiments have also found null results, including assessment of the effects of new supermarkets in food deserts [34, 75, 76] fast-food retail expansion [77] as well as regulation of calorie labeling [78] on diet. An assesement of sidewalk improvement found no significant increases in physical activity as measured by accelerometry [79]. Yet others have shown that improved physical activity resources *when used* were associated with improved physical activity [80].

Even with great care, studies looking at neighborhood change may have difficulty detecting effects. The study of neighbourhood investments is complex. Neighbourhoods are dynamic and even with a neighbourhood undergoing substantial investments (whether it be in housing, greenspace, retail or commercial), and even with a rigorous design following a cohort within both an intervention and control neighbourhood, it is unlikely that a comparison neighbourhood will remain unchanged. Changes in intervention neighbourhoods may also be less than anticipated. There were greenspace developments anticipated in the intervention neighborhood not shown in Fig. 1 or studied in our analysis because they did not come to fruition. The greenspace and park renovation plans for the Hill District intervention neighborhood were much more extensive than what was eventually implemented. It should also be noted that PA is a very difficult behavior to change, [81, 82] and neighbourhood investments, such as housing improvements also may include negative consequences such as construction-related disruption, relocation of residents, or changes in market rates which may make result in housing affordability issues, all which could have negative spillover effects.

Although investments did not influence the outcomes studied in the present analysis, residents may experience other benefits. In prior work conducted by our team, we found declines in food insecurity and fewer new diagnoses of high cholesterol and arthritis following the introduction of a full-service supermarket (one of the major investments included in this analysis) [83].

In addition to the noted limitations, our study had important strengths. These include our inclusion of an ancillary “distance from investment” analysis, objectively measured physical activity, and diverse self-report measures. There were some notable changes in the latter over time within each neighbourhood. These suggest that our failure to detect an effect of investment was not a function of weak or insensitive measurement or a lack of neighbourhood change. Finally, our study addresses many of the issues present in other studies (selection into neighbourhood, residents who leave, disruptions caused by forced moving) [84].

## Conclusions

Although we observed changes over time in some outcomes that we tracked, ultimately there were no significant changes related to investments above and beyond what we might have expected without those investments. Still, to move the field forward, we need additional opportunities especially to pursue natural experiment or quasi-experimental designs which will allow us to better identify and understand the way in which neighbourhood investments may ultimately impact residents’ health.
